# Epigenetic silencing of checkpoint with fork-head associated and ring finger gene expression in esophageal cancer

**DOI:** 10.3892/ol.2013.1677

**Published:** 2013-11-11

**Authors:** YOSHIHIRO SUZUKI, YOHEI MIYAGI, NORIO YUKAWA, YASUSHI RINO, MUNETAKA MASUDA

**Affiliations:** 1 Department of Surgery, Hiratsuka Kyosai Hospital, Hiratsuka, Kanagawa 254-8502, Japan; 2 Molecular Pathology and Genetics Division, Kanagawa Cancer Center, Yokohama, Kanagawa 241-0815, Japan; 3 Department of Surgery, Yokohama City University, Yokohama, Kanagawa 236-0004, Japan

**Keywords:** checkpoint with fork-head associated and ring finger, methylation-specific polymerase chain reaction, esophageal cancer

## Abstract

Checkpoint with fork-head associated and ring finger (CHFR) is a mitotic checkpoint gene with tumor-suppressor functions. Previous studies have described the hypermethylation of the CpG island in the promoter region as a key mechanism involved in silencing tumor suppressor genes. The epigenetic alterations regulating CHFR expression and the clinical significance of CHFR downregulation remain unclear. A total of 40 patients with esophageal squamous cell carcinoma who underwent primary resection were enrolled in this study. CHFR mRNA expression was quantified, followed by an evaluation of the methylation status using methylation-specific polymerase chain reaction (MSP) techniques in 29 patients. The correlation between CHFR expression and MSP status was then analyzed. In addition, the significance of CHFR expression was determined, with respect to clinicopathological features and overall survival. Aberrant hypermethylation of the CHFR gene was observed in 13 of 29 primary esophageal cancers. The CHFR expression levels of the methylated status samples was significantly lower than that of the unmethylated status samples (P=0.014). CHFR expression levels did not exhibit clinical significance with respect to the patient characteristics or overall survival. Hypermethylation of the CHFR gene is a common event in the development of primary esophageal cancer. CpG island hypermethylation of the promoter region in the CHFR gene is a key mechanism involved in silencing the CHFR gene in patients with esophageal cancer.

## Introduction

Esophageal cancer remains a major cause of cancer mortality worldwide. Patients with this cancer generally have a poor prognosis. Conducting detailed molecular and genetic investigations is necessary to develop new strategies for esophageal cancer.

Previous biological studies have shown that numerous types of cancer are caused by the accumulation of multiple genetic defects in dominant oncogenes and tumor suppressor genes involved in the regulation of the cell cycle, induction of apoptosis and modulation of checkpoints (1–4).

Checkpoint genes are well-known tumor suppressors. Their main functions are to stop the cell cycle and repair errors when cells are exposed to various stressors, thereby preventing DNA damage (5). Failure of these checkpoint functions results in genomic instability, a mutagenic condition that predisposes cells to neoplastic transformation (6,7).

Checkpoint with fork-head associated and ring finger (CHFR), a checkpoint gene, was first identified by Scolnick and Halazonetis in 2000 and described as a nuclear protein that plays an important role in the early mitotic stress caused by microtubule inhibitors (8). It has been reported that CHFR expression levels are downregulated in various digestive cancers, including esophageal cancer (9,10). However, the regulatory mechanisms and clinical significance of the CHFR gene remain unclear.

To date, a growing number of studies have reported that the loss of tumor suppressor genes is often caused by epigenetic alterations, including methylation of DNA (11,12). A number of studies have described hypermethylation of the CpG island in the promoter region as a key mechanism involved in gene regulation (13–15). However, the role of CpG island methylation in CHFR silencing in esophageal cancer has not been fully investigated.

The objective of the present study was to evaluate the correlation between CHFR expression and the methylation status of the CpG island in the promoter region. The significance of CHFR gene expression with respect to clinicopathological features and survival was also examined.

## Materials and methods

### Sample collection and DNA/RNA preparation

A total of 40 patients were enrolled in this study. The patients had primary esophageal squamous cell carcinoma and underwent primary resection at the Kanagawa Cancer Center (Yokohama, Japan) between November 2001 and July 2003. There were 36 males and 4 females, with a mean age of 64.8 years (45–76 years). The study was approved by the institutional review board committee of Kanagawa Cancer Center Hospital and written informed consent for the study was obtained from all patients. The tumor samples were obtained during surgical resection of esophageal cancer and immediately stored at −80°C. Genomic DNA was extracted from the tumor tissues using the DNA extraction kit, Sepa-Gene (Sanko-Junyaku, Tokyo, Japan). RNA was extracted from the tumor tissues using TRIzol reagent (Life Technologies, Tokyo, Japan).

### Reverse transcription polymerase chain reaction (RT-PCR)

Single-step RT-PCR was performed using CHFR gene-specific oligonucleotide primers for reverse transcription and PCR and fluorescent hybridization probes labeled with LC-Red 640 or FITC for real-time detection, using the LightCycler RNA Amplification kit HybriProbe and LightCycler (Roche Diagnostics, Tokyo, Japan). The housekeeping gene, human porphobilinogen deaminase (hPBGD), was simultaneously evaluated using to normalize the amount of RNA used in the reaction. The reaction was designed according to the manufacturer’s instructions without modifications and consisted of the following settings: 50°C for 10 min for reverse transcription and 95°C for 2 min for denaturation, followed by 45 cycles of 95°C for 1 sec, 55°C for 15 sec and 72°C for 10 sec. The same conditions were used for CHFR and hPBGD. The nucleotide sequences of the primers and hybriprobes are shown below. For CHFR: forward 5′-gcctggccccgttttgtgagc-3′, reverse 5′-gacgggatgttacggccactg-3′, hybriprobe LC-Red 640 5′-ggccgtaacatcccgtcctga-3′ and hybriprobe FITC 5′-attcctgcttccgagttgccag-3′ For hPBGD: forward 5′-accctgccagagaagagtgt-3′, reverse 5′-ccacagcatacatgcattcc-3′, hybriprobe LC-Red 640 5′-ctgaactccagatgcgggaactt-3′ and hybriprobe FITC 5′-ggtgttgaggtttccccgaatact-3′.

### Bisulfite modification

Genomic DNA obtained from the tumors was subjected to bisulfite modification. A total of 2 μg genomic DNA was incubated in 50 μl water and denatured in 0.2 M NaOH for 10 min at 37°C. The denatured DNA was then diluted in 550 μl of a solution containing 30 μl hydroquinone (10 mM) and 520 μl sodium bisulfite (3 M). The DNA solution was incubated for 16 h at 50°C. Following incubation, the DNA sample was desalted using the Wizard DNA Clean-Up System (Promega Corporation, Madison, WI, USA) and treated with 0.3 M NaOH for 5 min at room temperature. Finally, the DNA sample was precipitated with ethanol, dissolved in 32 μl TE buffer and stored at −20°C.

### Methylation-specific PCR (MSP)

MSP exploits the effects of sodium bisulfite on DNA, which efficiently converts unmethylated cytosine to uracil while leaving methylated cytosine intact (16). Consequently, following modification, the unmethylated and methylated alleles have different sequences that can be used to design allele-specific primers for PCR. The primer sequences for hCHFR have been described previously (16,17). The primers used for the unmethylated reaction were as follows: 5′-ata taa tat ggt gtt gat t-3′ (sense) and 5′-tca act aat cca caa aac a-3′ (antisense). The primers used for the methylated reaction were as follows: 5′-ata taa tat ggc gtc gat g-3′ (sense) and 5′-tca act aat ccg cga aac g-3′ (antisense). PCR amplification was performed in 20 μl reaction volumes containing 2.0 μl 10X PCR buffer, 0.5 μl *Taq* DNA Polymerase, 0.8 μl primer mixture (10 μM each) and 2.0 μl modified DNA. The annealing temperature was 50°C for the unmethylated samples and 58°C for the methylated samples. The PCR amplification conditions were as follows: 94°C for 2 min, 42 cycles of 94°C for 30 sec, the specific annealing temperature for 30 sec and 68°C for 1 min. The PCR products were 206 bp in size for each sample. The PCR products were subjected to gel electrophoresis through a 2% agarose gel, stained with ethidium bromide and then visualized under UV illumination.

### Statistical analyses

The statistical correlation between CHFR expression and MSP status was analyzed using the Mann-Whitney U test. The CHFR expression levels and patient characteristics, including age, gender, histological type, depth of invasion, lymph node metastasis, lymphatic invasion, venous invasion, pathological stage and intraluminal metastasis were compared using the χ^2^ test.

The post-operative survival rate was analyzed according to the Kaplan-Meier method, and differences in survival rates were assessed using the log-rank test.

All statistical analyses were conducted using the Dr. SPSS II software program, version 11.0.1J for Windows (SPSS, Inc., Chicago, IL, USA). P<0.05 was considered to indicate a statistically significant difference.

## Results

### RT-PCR

**Figure 1:**
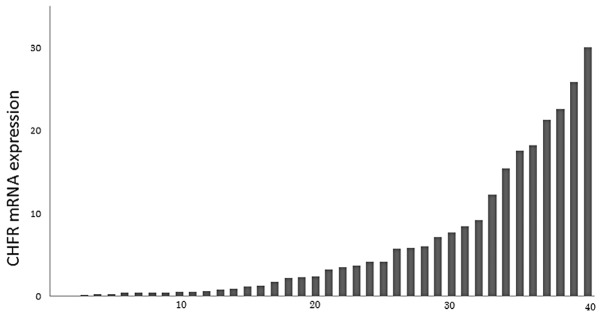
CHFR mRNA quantification with LightCycler in 40 esophageal cancer samples. CHFR, checkpoint with fork-head associated and ring finger.

Using RT-PCR, CHFR gene expression in 40 primary esophageal squamous cell carcinomas was quantified. The relative levels of CHFR mRNA expression are shown as a ratio of hPBGD expression. Esophageal cancers exhibited a variety of levels of CHFR gene expression (Fig. 1).

### MSP

**Figure 2:**
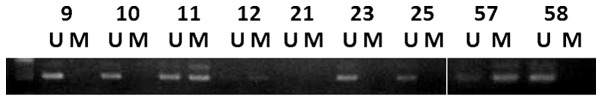
MSP analysis of DNA from esophageal cancer. In total, 14 of 31 cases showed marked hypermethylation of the CHFR promoter region. The concurrent amplification of methylated and unmethylated status was considered as methylated status. U, unmethylated DNA-specific amplification; M, methylated DNA-specific amplification. MSP, methylation-specific polymerase chain reaction; CHFR, checkpoint with fork-head associated and ring finger.

MSP was subsequently successfully performed in 29 cases. Therefore, the methylation status in 29 of 40 primary esophageal cancers was investigated using the MSP technique. Amplification of the methylated DNA-specific PCR primers was observed in 13 of 29 primary esophageal cancers (44.8%), while that of the unmethylated primers was observed in 16 patients (55.2%). Concurrent amplification of a methylated and unmethylated status was defined as a methylated status (Fig. 2).

### Correlation between the MSP status and CHFR gene expression levels

**Figure 3:**
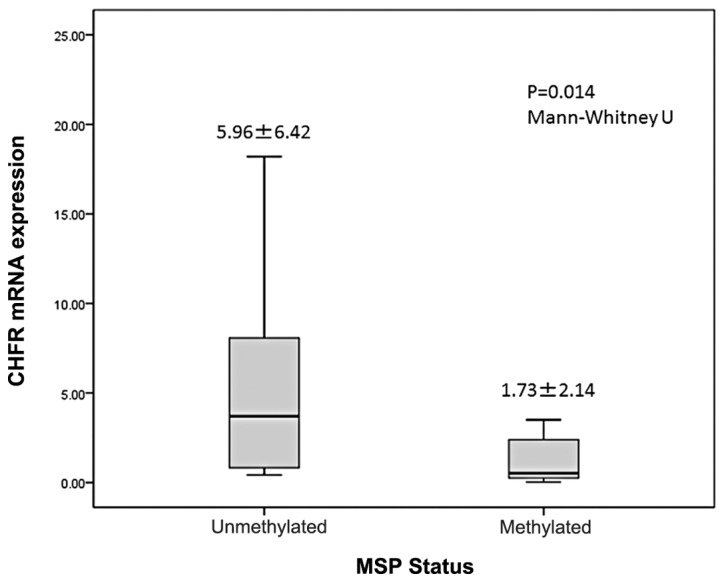
MSP status correlated significantly with CHFR gene expression levels in primary esophageal cancer. Box indicates the 75th and 25th percentile, horizontal line indicates the mean; bars indicate the 10th and 90th percentile. MSP, methylation-specific polymerase chain reaction; CHFR, checkpoint with fork-head associated and ring finger.

CHFR expression levels of the methylated status samples were significantly lower than that of the unmethylated status samples (1.735±2.149 vs. 5.966±6.429; P=0.014; Mann-Whitney U test; Fig. 3).

### Correlation between CHFR gene expression levels and clinicopathological features

The expression levels of the CHFR gene were categorized as low or high according to the median value. The correlation between the expression levels of this gene and clinicopathological features was then examined. None of the clinicopathological features were found to correlate with CHFR expression levels (Table I).

### Correlation between CHFR gene expression levels and survival

**Figure 4:**
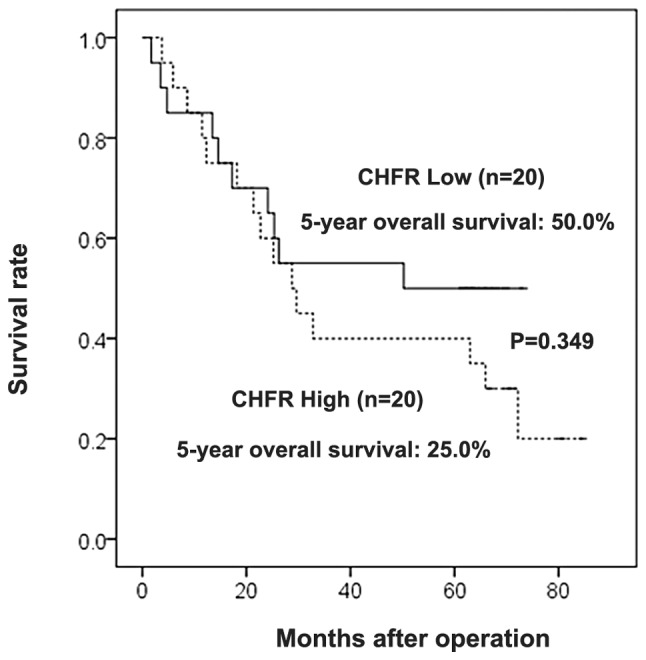
Expression levels of the CHFR gene did not correlate with survival in esophageal cancer (P=0.349). CHFR, checkpoint with fork-head associated and ring finger.

In the current study, the median follow-up period was 50.3 months. The overall survival rates were not significantly correlated with the CHFR expression levels (CHFR-high, 25.0%; CHFR-low, 50.0%; P=0.349, log-rank test) (Fig. 4).

## Discussion

Checkpoint genes, one of the surveillance mechanisms of cells, act to maintain genomic stability against various types of damage to the genome. The G1 checkpoint prevents replication of damaged DNA, while genomic integrity prior to mitosis is monitored by the G2 checkpoint, which promotes G2 arrest on detection of DNA damage. As such, checkpoints are important for preventing the propagation of cells with corrupted genomes that could potentially cause tumor formation.

CHFR, a mitotic checkpoint gene, has been previously cloned and is localized to chromosome 12q24. It has been reported that the CHFR protein delays entry into mitosis at the G2 to M entry site (8,18–20). In fact, CHFR knockout mice are cancer-prone (21), indicating that the CHFR gene functions as a tumor suppressor. Previous studies have revealed that CHFR expression levels are frequently downregulated in patients with digestive cancer, including esophageal cancer (22,23).

The epigenetically-mediated loss-of-gene function is a well-known mechanism involved in carcinogenesis (24). Several tumor suppressor genes containing CpG islands can be silenced via methylation of the CpG island. Previously, aberrant methylation of the CHFR gene associated with gene silencing has been demonstrated in several studies (11,25), although it has not been fully clarified how the CHFR gene is regulated in esophageal cancer.

In the present study, mRNA expression of CHFR was assessed in 40 primary tumor samples using RT-PCR, which showed a variety of CHFR expression levels. Then, MSP was carried out to evaluate the methylation status of CpG islands in the promoter region of the CHFR gene in 29 cases. The results indicate that aberrant methylation of the CHFR gene is frequently (44.8%) observed in esophageal cancer. This frequency is higher than that reported in previous studies, which have described a frequency of 16.3–24.0% (11,26,27). The differences in this rate may be due to disparities in the primer design, resulting in different degrees of CpG amplification.

In the present study, downregulation mechanisms were analyzed, revealing that aberrant methylation of the promoter region correlated with a loss of CHFR mRNA expression. Shibata *et al*(27) also confirmed the presence of a significant correlation between CHFR methylation and downregulation, which supports our previous results. Other data have shown that the interplay between epigenetic and genetic mechanisms underlies the loss of CHFR function in esophageal adenocarcinoma (10), indicating that there are various steps involved in suppressing mRNA expression of the CHFR gene.

Subsequently, the effect of CHFR gene expression levels on clinicopathological features was evaluated, and no correlation was found. Our results also revealed that CHFR expression levels do not have prognostic significance in patients with esophageal cancer. The clinical importance of CHFR expression in esophageal cancer is not well studied. To date, a single study has been performed to investigate the correlation between CHFR expression and patient characteristics in esophageal cancer, and indicated no relative clinical factors (27). These results indicate the possibility that CHFR silencing is associated with carcinogenesis, without tumor progression.

Taken together, results of the present study indicate that aberrant hypermethylation of CpG islands is the key mechanism associated with transcriptional inactivation of the CHFR gene in patients with esophageal cancer. It has been previously shown that it is possible to reverse epigenetic changes and restore gene function using treatment with DNA methylation inhibitors (28). Further investigations are likely to provide new insights into establishing novel strategies for treating esophageal cancer.

## Figures and Tables

**Table I:** Correlation between expression of the CHFR gene and clinicopathological features.

	CHFR expression		
		
Variables/Categories	Low (n=20)	High (n=20)	P-value
Age	67.0±5.26	62.7±6.86	0.156
Gender			
Male	18	18	0.698
Female	2	2	
Histological type			
Well-differentiated	4	3	0.264
Moderately-differentiated	8	6	
Poorly-differentiated	8	11	
Depth of invasion			
T1	1	2	0.493
T2	4	12	
T3	15	15	
T4	0	1	
Lymph node metastasis			
Absent	3	5	0.376
Present	17	15	
Number of lymph node metastasis			
0–3	15	11	0.347
≥4	5	9	
Lymphatic invasion			
Absent	9	11	0.376
Present	11	9	
Venous invasion			
Absent	2	3	0.500
Present	18	17	
Stage			
I	0	1	0.599
II	5	4	
III	14	13	
IV	1	2	
Intraluminal metastasis			
Absent	18	18	0.698
Present	2	2	

CHFR, checkpoint with fork-head associated and ring finger.
